# Tools for studying and modulating (cardiac muscle) cell mechanics and mechanosensing across the scales

**DOI:** 10.1007/s12551-021-00837-2

**Published:** 2021-09-05

**Authors:** Pamela Swiatlowska, Thomas Iskratsch

**Affiliations:** School of Engineering and Materials Science, Queen Mary University of London, London, UK

**Keywords:** Mechanobiology, Force, Cardiomyocytes, Cardiac tissue, Scale, Tools

## Abstract

Cardiomyocytes generate force for the contraction of the heart to pump blood into the lungs and body. At the same time, they are exquisitely tuned to the mechanical environment and react to e.g. changes in cell and extracellular matrix stiffness or altered stretching due to reduced ejection fraction in heart disease, by adapting their cytoskeleton, force generation and cell mechanics. Both mechanical sensing and cell mechanical adaptations are multiscale processes. Receptor interactions with the extracellular matrix at the nanoscale will lead to clustering of receptors and modification of the cytoskeleton. This in turn alters mechanosensing, force generation, cell and nuclear stiffness and viscoelasticity at the microscale. Further, this affects cell shape, orientation, maturation and tissue integration at the microscale to macroscale. A variety of tools have been developed and adapted to measure cardiomyocyte receptor-ligand interactions and forces or mechanics at the different ranges, resulting in a wealth of new information about cardiomyocyte mechanobiology. Here, we take stock at the different tools for exploring cardiomyocyte mechanosensing and cell mechanics at the different scales from the nanoscale to microscale and macroscale.

## Introduction

**Fig. 1:**
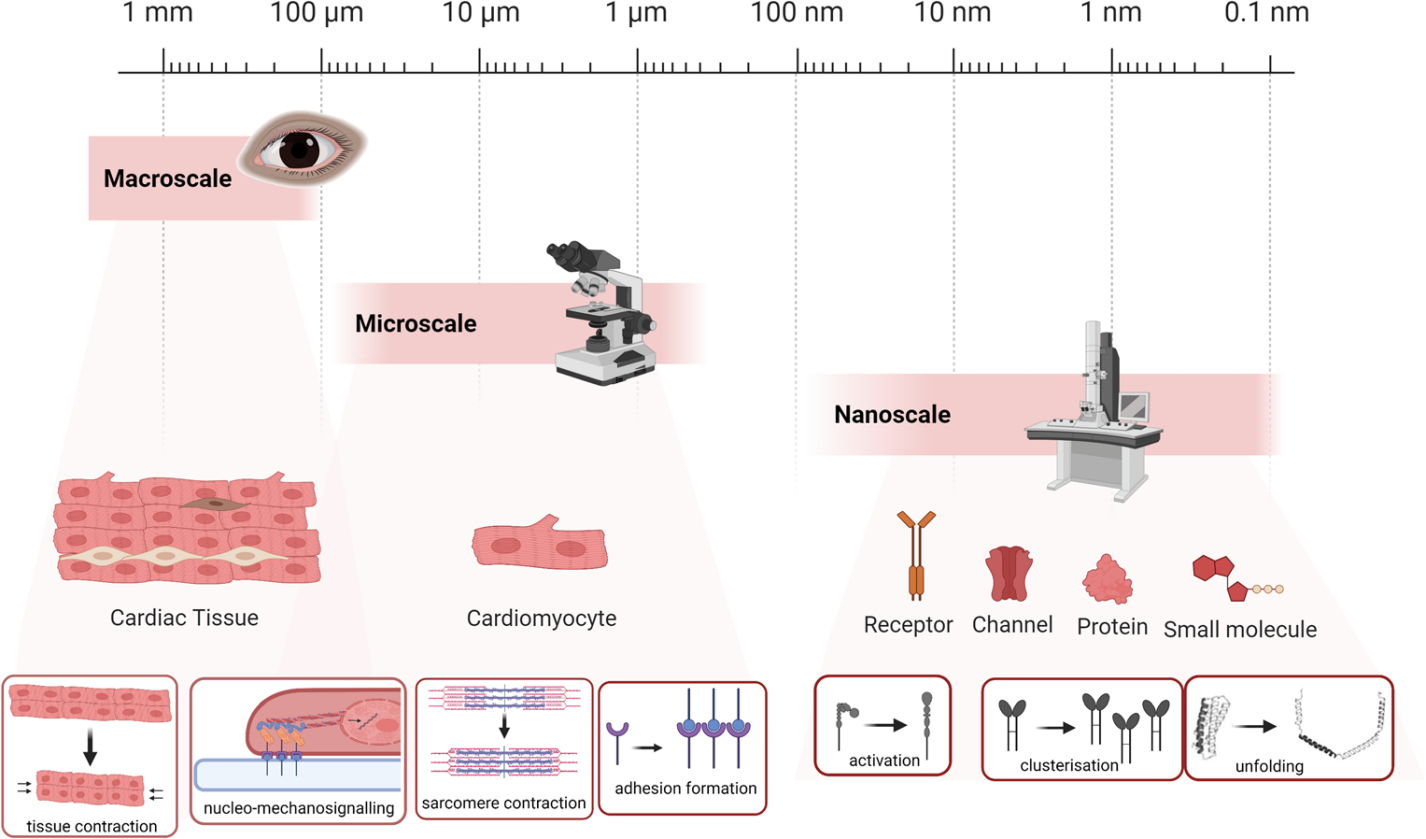
Studying cardiac biology across the scales. Nanoscale platforms enable the study of forces and mechanosensitive dynamics at single molecule, single adhesion or single adhesion cluster level that effectively cause protein activation, cluster formation or protein (domain) unfolding. Microscale and macroscale tools allow studying intracellular changes, the cell–cell or cell–ECM cross-talk

It is now clear that mechano-regulation is an integral part of cells and tissues in both physiological and pathological conditions (Beedle et al. 2017; Rivas-Pardo et al. 2016). Along with the recognition of mechanobiology as an indispensable partner of biological samples studies, new techniques emerge to address this topic. The advent of new tools advanced our fundamental knowledge of mechano-regulation in a range of cell and tissue types. Likewise, understanding mechanosensing and transduction processes is pivotal for explaining cardiovascular physiology and pathology (Sit et al. 2019; Ward and Iskratsch 2020). The heart is experiencing different types of forces such as shear stress and tensile force (Lu and Kassab 2011; Lunkenheimer et al. 2004). Structural and mechanical hierarchies span from nanoscale to macroscale. The interconnection through the extracellular matrix leads to sensing of macroscale forces at specific mechanosensor molecules, which again leads to mechanical control of cell fate switching and tissue development (Ingber 2008). Most (mechanobiology) studies focus on a single scale, and mechanical measurements of single myofibrils, cardiomyocytes, trabeculae or cardiac tissue individually shed more light on the overall cardiac mechanobiology (Brady et al. 1979; Carson et al. 2016; Kim et al. 2006; Saleem et al. 2020). Here, we allocated various approaches that map cardiac mechanical alterations based on the scale of operation, nano, micro and macro (Fig. 1). We will progressively introduce tools in their respective range of action, subsequently illustrating the obtained outcome. This review will disseminate the different techniques and newest data that have been gained from these studies. Work carried out at different scales and by different techniques resulted in confounding results every so often (examples are the different mechanical measurements of the cardiac stiffness, different forces necessary to unfold monomers or higher ordered structures, such as filaments or fibrils, or different types of forces measured with nano vs micropillars) (Ghassemi et al. 2012; Meacci et al. 2016; Roca-Cusachs et al. 2012; Ward and Iskratsch 2020). Therefore, it is becoming ever clearer that approaches, models and theories are needed that bridge the scales in cardiovascular mechanobiology and mechanobiology in general (Regazzoni et al. 2020). In the last paragraph, we will discuss the challenges and future perspectives for the cardiac mechanobiology techniques, including attempts to integrate the data from the different scales for an improved understanding of cardiomyocyte mechanical sensing.

## Cardiovascular mechanosensing at the nanoscale: from single molecules to single adhesions

**Fig. 2:**
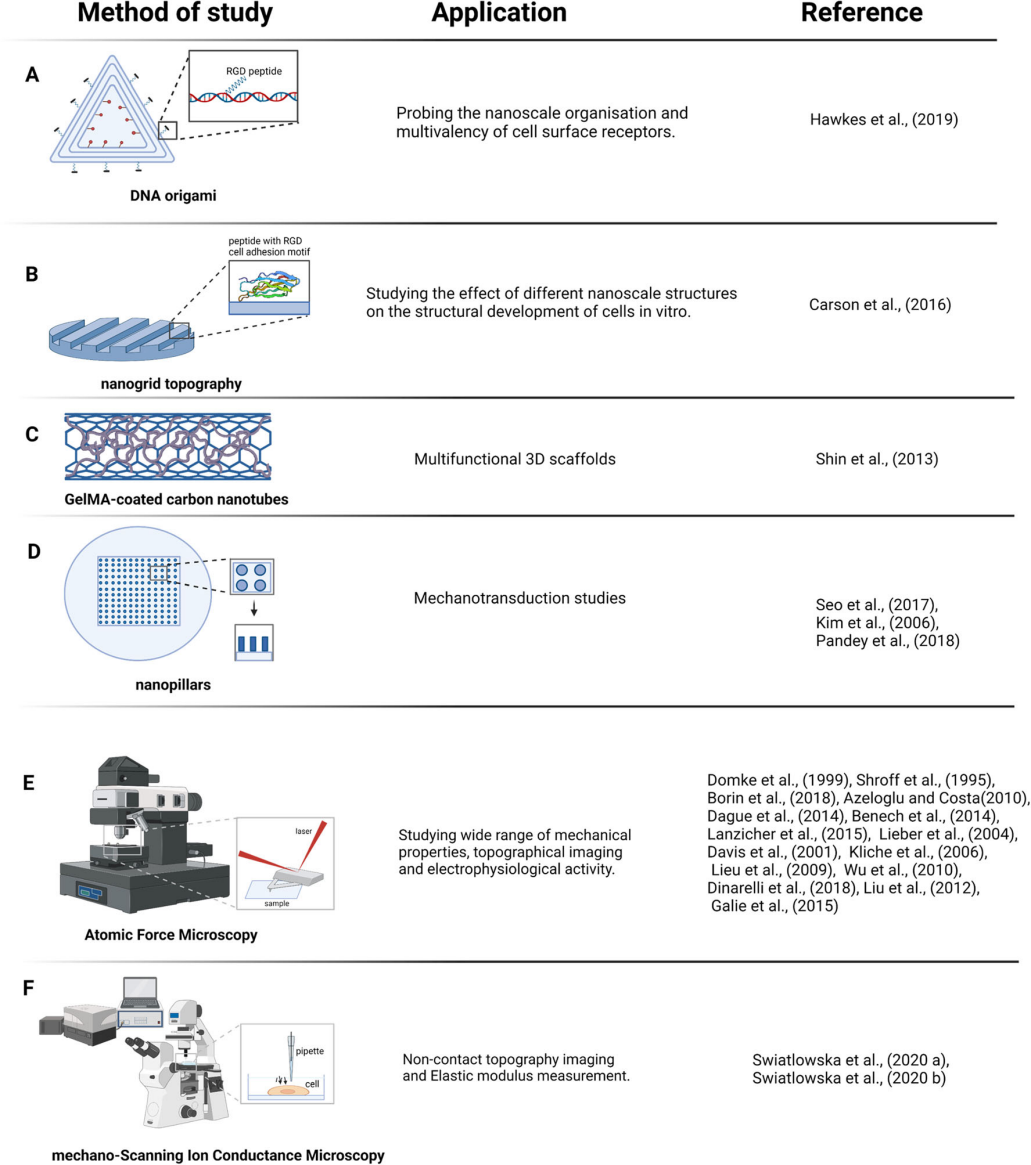
Selected nanoscale tools that have been employed for the investigation of cardiovascular mechanobiology (first column), as well as their applications (second column) and respective references (third column). (**A**) DNA origami, (**B**) nanogrid topography, (**C**) gelatin methacryloyl (GelMa)-coated carbon nanotubes, (**D**) nanopillars, (**E**) atomic force microscopy and (**F**) mechano-scanning ion conductance microscopy. The increasing number of available tools, as well as the number of studies using these tools, reflects the importance of the processes happening at the nanoscale

In recent years, a surge in new nanoscale approaches can be observed that aim to study forces and mechanosensitive dynamics at single molecule, single adhesion or single adhesion cluster level (Fig. 2). Despite the dimensions, the final effect can be likewise observed at a bigger scale (Sanchez-Alonso et al. 2020). These approaches include studies applying forces at single molecules using magnetic tweezers, laser traps or atomic force microscopes; or measurement of forces and protein dynamics at the single adhesion level using nanofabricated tools such as nanopillars or nanopatterns. Other nanofabrication techniques are being employed to study the effect of topography or ligand presentation on cell behaviour (nanopatterns, nanofibers, nanotubes or nanowires).

### Single-molecule studies — magnetic tweezers, optical tweezers and atomic force microscopy (AFM)

Applying forces onto single molecules gives insights into nanoscale mechanics, including force-dependent domain folding and unfolding events, opening of catalytic domains or cryptic binding sites that change protein–protein interactions and ultimately determine mechanosensitive responses at the cellular scale. The main techniques in general and especially for cardiovascular mechanobiology are AFM, magnetic and optical tweezers. The details of the techniques have been discussed elsewhere (e.g. by Neuman and Nagy 2008) and are out of scope for this short review, but briefly, all techniques measure force-dependent change in length of a single molecule that is attached to either the tip of the cantilever (AFM) or a bead (magnetic tweezer, optical tweezer) on one side and a solid surface or another bead on the other end. Depending on the technique, forces are applied through a piezo controlled cantilever (AFM), a magnetic field on paramagnetic beads (magnetic tweezers), or a focused laser beam onto a dielectric bead (optical tweezers) (Neuman and Nagy 2008).

Notable studies that are pertinent for the cardiovascular field are especially experiments exploring the mechanics of titin, which is a major contributor to cardiomyocyte mechanics. Here, recent studies investigated the unfolding and the refolding of titin domains that could contribute to the force generation in muscle cells (Mártonfalvi et al. 2017; Rivas-Pardo et al. 2016), although the relevance for cardiac muscle might be limited due to much smaller amount of force generated through this mechanism, compared to muscle myosin contractions, as well as a 60-fold slower shortening velocity (Bianco et al. 2016). Other single-molecule studies on titin observed the modulation of titin stiffness through oxidative folding (Beedle et al. 2017) or mechanical activation of ATP binding to the titin kinase domain (Puchner et al. 2008), albeit this has been since identified as a pseudokinase, which nonetheless is involved in scaffolding processes of regulatory significance (Bogomolovas et al. 2014; Lange et al. 2005).

Looking at the cell-matrix interface, especially two molecules of relevance have been studied using such single-molecule approaches: talin and dystrophin (del Rio et al. 2009; Haining et al. 2018; Le et al. 2018; Yao et al. 2016). Talin was first identified to unfold cryptic binding sites to enable vinculin binding (del Rio et al. 2009) and later also to regulate the interaction with the Rho GTPase activating protein DLC1 (Haining et al. 2018) and potentially additional binding partners (Yao et al. 2016). Recently, magnetic tweezer studies suggested dystrophin, which is the central component of the dystrophin–glycoprotein complex that links the extracellular matrix with the actin cytoskeleton, to act as molecular shock adsorber through force-dependent unfolding and refolding of its spectrin domains (Le et al. 2018). Overall, these studies reinforce the idea that cells such as cardiomyocytes are finely tuned to a specific mechanical environment and use mechanosensitive proteins to react to small changes in the force landscape.

### Measuring cellular forces at the nanoscale — nanopillars

Nanopillars, typically fabricated by using e-beam lithography to make a negative master, followed by PDMS soft lithography, have been shown to be a very practical tool in the mechanical characterization of different cell types, including cardiomyocytes. These can at the same time mimic substrate stiffness — typically modulated by adjusting the pillar aspect ratio — and be used to measure the cellular forces. Together, this allows obtaining detailed information of cell–ECM interactions and cell mechanics. Compared to micropillars, nanopillars are recognised by cells as uniform surfaces and fibroblast adhesions spread over multiple pillars instead of forming around individual posts. Therefore, nanopillars can be used to pick up forces that are generated e.g. during early adhesion formation (Iskratsch et al. 2013; Wolfenson et al. 2014; Haguy Wolfenson et al. 2015). Applied to a study of cardiovascular mechanosensing, our previous work could demonstrate cardiomyocyte rigidity sensing depending on slow non-muscle myosin and fast muscle myosin contractions. This results in oscillating stretching of talin protein on physiological substrates but continuous stretching on fibrotic stiffness (Pandey et al. 2018). Other groups used nanopillars to further study the effect on cardiomyocyte differentiation and behaviour. Nanoscaled gradient pillar patterned plates used by Seo et al. increased cardiomyocyte differentiation, showing highly organised sarcomere formation and mature cardiac gene expression on 200–280 nm-sized pillars. This process was associated with phospho-cofilin mediated actin cytoskeleton reorganisation (Seo et al. 2017). Nanopillar platforms were also applied to neonatal rat cardiomyocytes cultures. Poly(ethylene glycol) (PEG) hydrogel pillars have shown lower cell adhesion but higher action potential amplitude (Kim et al. 2006).

Contact points between the cell and extracellular matrix serve as anchoring points and mechanotransduction nodes activating further signalling pathways. The cell mechanosensing is influenced not only by stiffness but also by the type and organisation of the extracellular matrix molecules or respective receptors and e.g. different integrins show different mechanosensing behaviours (Elosegui-Artola et al. 2014; Schvartzman et al. 2011; Ward and Iskratsch 2020). To study the organisation of receptors, for instance integrins, DNA origami nanoarrays functionalized with peptides have been employed to examine cardiomyocyte–ECM interaction at the single receptor (cluster) level. Studies on neonatal rat cardiomyocytes demonstrated that both the distance between individual integrin ligands and the density of the ligands influence cardiomyocyte adhesion — in contrast to fibroblasts which only respond to the inter-ligand distance (Hawkes et al. 2019). Since costameres connect the cytoskeleton to the ECM not only through integrins and associated proteins, but also through the dystrophin–glycoprotein complex (DGC), it will be intriguing to expand these studies onto different adhesion systems or to study the cross-talk between the receptors as done e.g. between EGF and integrins in cancer cells (Huang et al. 2018).

### Measuring cardiomyocyte mechanics at the nanoscale — AFM and scanning ion conductance microscopy (SICM)

#### AFM

Application of atomic force microscopy (AFM) for biological sample studies, including cardiomyocytes, started in the 90s. First reports showed a link between regional cell stiffness to changes in the cytoskeleton. Moreover, AFM was employed to measure cell contractile activity by following the cantilever deflection over time with nanoscale resolution (Domke et al. 1999; Shroff et al. 1995). These first studies sparked interest in this method and inspired researchers to use it for a range of different research questions. To date, AFM has been employed to study cardiomyocyte properties, such as topographical changes, contraction activity and mechanics (Borin et al. 2018). The elastic modulus was assessed in different states (diastole and systole) to assess chemically induced cellular changes, effects of knockouts or disease-causing mutations. These studies pointed out key proteins that regulate the elastic modulus, such as actin and vinculin, or whole membrane microdomains (Azeloglu and Costa 2010; Benech et al. 2014; Dague et al. 2014; Lanzicher et al. 2015). Using AFM, age was also shown to be an important determinant of the elastic modulus, whereby cardiomyocytes isolated from 30-month-old rats were stiffer than cells from 4-month-old animals, suggesting that change in single cardiomyocyte mechanical properties can also contribute to left ventricular diastolic dysfunction observed in elder patients (Lieber et al. 2004).

AFM as an imaging tool was applied to resolve high-resolution sarcolemma features in adult guinea pig cardiomyocytes (Davis et al. 2001), aldosterone-mediated sarcolemma changes in mouse neonatal cardiomyocytes (Kliche et al. 2006) and used to demonstrate lack of t-tubular cardiomyocyte membrane invaginations in mouse and human embryonic stem cell-derived cardiomyocytes (ESC-CMs) (Lieu et al. 2009). Moreover, since the cardiomyocyte membrane presents a rich repertoire of different receptors that play a major role in the overall cell physiology, coating the AFM tip with specific protein allowed to measure adhesion forces that were shown to be disrupted in mutant cardiomyocytes (Lanzicher et al. 2015; Wu et al. 2010).

When AFM was used to measure contraction dynamics of hiPSC-derived cardiomyocytes from control and myotonic dystrophy type 1 patients, a higher mechanical resistance was observed based on altered beating impulse or beat duration (Dinarelli et al. 2018). Similarly, a multi-parameter AFM-based study measured contraction force, rate and duration as well as elastic modulus in hESC-CM and iPSC-CM, allowing to map spatial heterogeneity of height, elastic modulus and contraction force, together demonstrating aberrant contractility and mechanical properties in iPSC–cardiomyocytes from dilated cardiomyopathy patients (DCM) patients (Liu et al. 2012). Also, AFM was also used to stimulate cell contraction in a cardiac microtissue in vitro (Galie et al. 2015), by applying oscillating indentations to train the cells. Further modifications to the system present the possibility to study depolarization and repolarization wavefronts.

#### SICM

A non-contact, high-resolution mechano-scanning ion conductance microscopy (mechano-SICM) technique, measuring the transverse Young’s modulus (tYM) by a constant pressure application through a nanopipette was recently used to investigate specific membrane subdomains, enabled by the excellent resolution in all dimensions (Swiatlowska et al. 2020a; Swiatlowska et al. 2020b). When applying pressure, the probe’s vertical position is recorded, and a deformation map is generated from which the tYM can be calculated and corrected for the uneven geometry. Both tYM and topography maps are recorded simultaneously and non-invasively, leaving the cell intact in non-contact mode. This work demonstrated increased tYM in cardiomyocytes from a myocardial infarction (MI) rat model, where the mechanical load is high compared to control animals. On the other hand, in cardiomyocytes from a load-deficient MI model, the tYM was reduced. Observed changes were due to an altered microtubular network that has also been shown to regulate the modified tYM in the Angiotensin II-treated adult rat cardiomyocytes (Swiatlowska et al. 2020a; Swiatlowska et al. 2020b). The high resolution and non-invasive nature of the contactless scanning mode are major benefits of this platform, suggesting great potential for expanding the use to other mechano-regulatory cell measurements.

### Nanotopography sensing and tools for controlling cardiomyocyte differentiation and behaviour

Different tools have been employed to study topography sensing at the nanoscale. Fundamental knowledge of the topography sensing mechanisms has further influenced the design of nanotopographies that mimic the native tissue in vitro and thus improve the maturity and function of cardiomyocytes. These include nanofibers or nanowires.

#### Nanofibers

Obtaining induced pluripotent stem cell-derived cardiomyocytes (iPSC-CM) functionally and structurally similar to adult cardiomyocytes is still very challenging. Moreover, one of the overlooked aspects in graft implantation is the maintenance of cell directionality. If poorly performed, region-specific alterations are observed, and myofiber orientation differences between the transplant and diseased tissue occur. However, carefully designed topographies can be harnessed for improved maturation. This was shown for instance when iPSC-CM were plated on nanogrooved topographies, which were mimicking ECM fibre orientation and were functionalised with RGD as cell adhesive peptides (Carson et al. 2016). Morphological analysis of cell alignment and area, as well as sarcomere length, suggested that cells plated on 800-nm diameter grooves improved maturation of the cardiomyocytes and showed the strongest resemblance to adult-like phenotypes among all nanotopographic patterns that were studied (Carson et al. 2016). In order to recapitulate the extracellular matrix (ECM) organisational structure, Lin et al. (2014) produced aligned and randomly oriented electrospun patches. Cardiomyocytes plated on aligned substrates demonstrated improved beating capabilities and, after patching onto infarcted hearts, showed significantly enhanced performance in vivo, as measured by improved hemodynamics, electrocardiography, optical mapping or reduced infarct size, as well as demonstrated by cell morphology with aligned anisotropic cardiomyocytes present after implantation; all are important features for therapeutic application (Lin et al. 2014). Similarly, when aligned nanofiber scaffolds were used to mimic ECM organisation, iPSC-cardiac progenitor cells showed improved cardiac maturation as shown by increased cTnt-positive cells, elongated nuclei and synchronised Ca2+ fluctuations (Ding et al. 2020).

#### Nanotubes

The advent of carbon nanotubes (CNTs) sparked a lot of interest in the biological field, opening doors for new cross-discipline studies. These 1–100 nm diameter graphene, cylindrically shaped structures offer good mechanical, electrical and thermal properties that enabled to use them in scaffolds in different forms. Although typically not used to modify the topography but rather for electrical excitation or sensing, it is noted in several studies that cardiomyocytes interact specifically with the nanotubes, forming a nanofibrous network (Martinelli et al. 2013b; Shin et al. 2013), and hence, this is discussed here as well. Studies on neonatal cardiomyocytes show that CNT substrates had a positive outcome on cell physiology, presenting higher cell viability, proliferation, tighter cell–cell contacts and improved electrophysiological parameters (Martinelli et al. 2013b). In a different study from the same group, CNTs also showed a protective effect from pathological hypertrophy by demonstrating no change in the gene expression profiles following phenylephrine stimulation. Together, this work illustrates the higher maturity of cardiomyocytes and disease preventive properties of CNTs (Gerwig et al. 2012; Martinelli et al. 2013a; Martinelli et al. 2013b). Also, an improvement towards cardiomyocyte lineage differentiation from mesenchymal stem cells (MSCs) was observed using CNT platforms. Here, by taking the advantage of the electrical properties, stimulated MSCs re-oriented and showed elongated morphology. Additionally, an increase in GATA-4, Nkx2.5, connexin43 and cardiac troponin T was observed (Mooney et al. 2012). To improve biocompatibility, biodegradability, electrical and mechanical properties, a CNT platform was developed, in which the nanostructures were homogenously embedded in gelatin methacrylate (GelMA) hydrogels with highly porous structures for tissue development. This new platform demonstrated improved cell adhesion, organisation, cell–cell coupling, mechanical integrity and electrophysiological properties in neonatal rat cardiomyocytes when compared to GelMA only arrays (Shin et al. 2013). Also, a protective effect from doxorubicin and heptanol was detected, together demonstrating the potential of CNT containing scaffolds for cardiovascular applications.

#### Nanowires

By looking at the specific nature of myocardial tissue architecture, it is noticeable that cellular orientation in the tissue is not random. Different techniques have been employed to address cardiomyocyte alignment in vitro cultures. Laser-patterned linear nanowires resulted in human cardiomyocyte alignment (Kiefer et al. 2014). In another study, fabricated gold nanowires (AuNWs) were incorporated in hydrogels, such as alginate or GelMA. Both studies showed that neonatal rat cardiomyocytes had a more mature state when cultured on NW hydrogels versus hydrogels alone (Dvir et al. 2011; Li et al. 2020).

### Translational approaches based on nanofabrication

Nanoscale techniques have been increasingly applied for drug testing purposes. Hart et al. generated a new in vitro platform that employed nanostructured interdigitated electrodes (nIDEs) patterned on polyacrylonitrile. The long-term culture of iPSC cardiomyocytes demonstrated that these nanopatterned arrays are highly sensitive and suitable for cardiotoxicity testing (Hart et al. 2018). On the other hand, inotropic and chronotropic drug effects were tested using AFM contractility measurements (Chang et al. 2013; Liu et al. 2012). The same technique was used to demonstrate an increase in membrane roughness in neonatal rat cardiomyocytes, following a hypertrophy protective drug uptake (Yang et al. 2013).

## Microscale tools

At the microscale, cardiomyocytes apply forces onto (and sense forces from) neighbouring cardiomyocytes and the extracellular matrix. Notably, the maturity and function of cardiomyocytes are affected at this scale by cell shape and extracellular matrix elasticity; hence, tools have been developed and applied to mimic and correlate between these healthy and diseased hearts. Early studies by the Discher lab established a clear correlation between cell differentiation and function with the substrate elasticity of polyacrylamide gels, whereby myogenic differentiation of MSCs and optimal work of quail cardiomyocytes were observed at a stiffness found in the native heart (~10 kPa) (Engler et al. 2004, 2008). Subsequently, similar results were obtained with a range of other materials and fabrication techniques, including PDMS, poly-e-caprolactone or PEG (Forte et al. 2012; Pandey et al. 2018; Wan et al. 2019), whereby the latter was also used to pattern the surface at the same time and found that patterning and stiffness together affected gene expression. Similarly, micropatterning was used to look at both healthy and fibrotic elastic moduli in combination with cardiomyocyte shape (i.e. aspect ratios of cardiomyocytes in healthy hearts, ~ 7:1; hypertrophic hearts, ~ 5:1; or dilated hearts, ~ 11:1), which suggests that the ECM elastic modulus regulates cardiomyocyte shape in order to obtain the most efficient contractile properties (McCain et al. 2014). Physiological shape and stiffness (10 kPa) were also shown to improve the cellular function of hPSC-CM. Defects in the myofibrils accumulated when increasing the substrates Young’s modulus, while keeping the aspect ratio constant at 7:1 (Ribeiro et al. 2015). Consequently, using patterned surfaces that impose biophysical cues such as geometrical constraint in 2D or 3D (eventually regulating cell shape) and substrate stiffness demonstrates a suitable approach to increase cardiomyocyte maturation and improve electrophysiological properties (Guo and Pu 2020).

Compared to nanopillars (see previous text), micropillars have been more widely used to study the mechanobiology of cardiomyocytes at different developmental stages. Micropost arrays coated with different ECM proteins showed no difference in contractile properties of hiPSC-CMs between laminin, fibronectin and collagen coatings. However, higher contractile properties were observed after thyroid hormone T3 treatment (Beussman et al. 2016; Rodriguez et al. 2014). HiPSC-CM cultured on micropillars exhibited different beating frequencies, elastic modulus, calcium signalling and sarcomere and integrin organisation compared to planar substrates (Palankar et al. 2016). Also, a more mature state of cultured neonatal rat myocytes was observed when these were cultured on microposts, inferred from higher twitch force, correlating with improved sarcomere organisation and intracellular calcium (Rodriguez et al. 2011).

Equally important to cell–ECM interaction is the cell–cell communication that includes a mechano-electrical activity. Using controlled substrate deformation similar to those exerted by neighbouring cardiomyocytes, microenvironment mechanical properties influenced cell mechanical coupling and hence led to synchronisation of the electrical functionality (Nitsan et al. 2016). Similarly, neonatal cardiomyocyte pairs on micropatterned surfaces demonstrated regularly repeating Ca2+ transients. These homogeneous cardiomyocyte pairs formed high traction stresses that were distributed at the lateral ends of the islands. In contrast, heterogeneous cell pairs of neonatal cardiomyocytes and stem cell-derived cardiomyocytes resulted in a tension variability with region-specific differences, with additional high stresses at the cell–cell junction (Aratyn-Schaus et al. 2016).

Micropatterned fibronectin islands on hydrogel supports were further used for the formation of multicellular ‘mini-tissues’, which showed stiffness dependent differences (Pasqualini et al. 2018). Cells on soft hydrogels (1 kPa) demonstrated shorter sarcomeres length and lower myofibrillar packing density and consequentially generated reduced contractile stresses in traction force microscopy (TFM) measurements when compared to tissues on normal (13 kPa) and stiff surfaces (90 kPa). Similar to previous observations (Engler et al. 2008), stresses increased with stiffness, while maximal contractile work was observed on normal gel stiffness (Pasqualini et al. 2018). Interestingly, metabolic measurements of basal respiration and ATP production suggested an inverse correlation with stiffness, while the spare respiratory capacity was independent of stiffness, suggesting that the additional available ATP was used for non-contractile purposes, such as cytoskeletal maturation.

In addition to TFM, intracellular and extracellular Förster resonance energy transfer (FRET) tension sensors have gained popularity, including cardiovascular research (Pandey et al. 2018). These are included here as microscale tools, due to optical limitations to the resolution for the detection of the forces, although super-resolution microscopy has been recently employed to surpass these limitations (Schlichthaerle et al. 2021). FRET tension sensors build on DNA double strands, DNA hairpins (both for extracellular application) or elastic peptides (for intracellular sensors). These force-sensitive elements are flanked by fluorescent molecules or quenchers that can be used to quantify the energy transfer as a function of the distance and after calibration with e.g. magnetic tweezers, as function of force (recently reviewed together with TFM and other tools in Lavrenyuk et al. 2021). Especially the intracellular tension sensors are promising tools to study poorly understood cardiomyocyte Z-disc, M-band or intercalated discs mechanobiology.

## Micro to macro

**Fig. 3:**
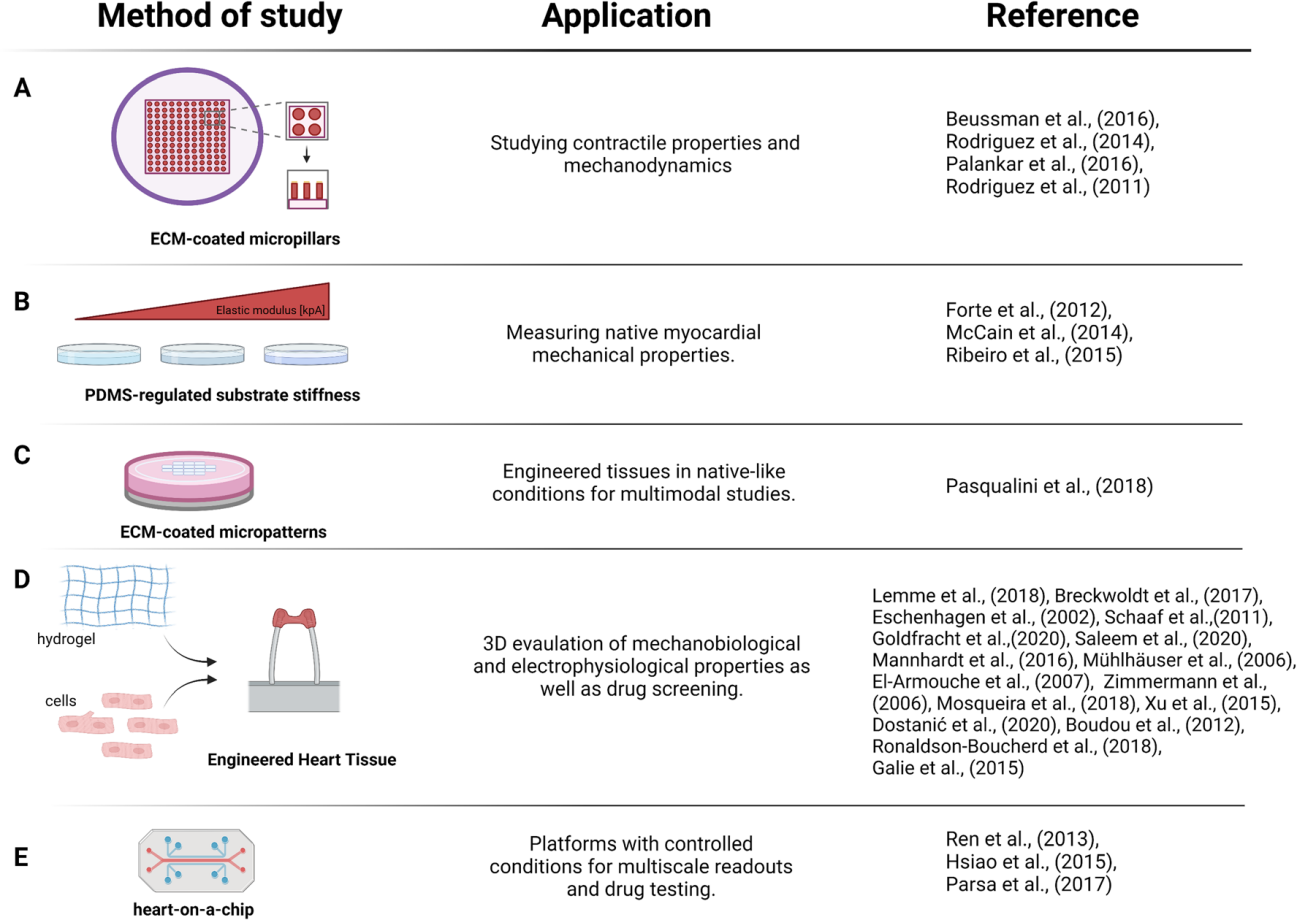
Selected micro and macro tools that have been employed for the investigation of cardiovascular mechanobiology (first column), as well as their applications (second column) and respective references (third column). (**A**) ECM-coated micropillars, (**B**) PDMS substrates, (**C**) ECM-coated micropatterns, (**D**) engineered heart tissues and (**E**) heart on a chip. Microscale and macroscale models have allowed the investigation of complex multicellular behaviours, mechanics and mechanobiology

Employing microscale and nanoscale tools greatly improved our knowledge of cardiomyocyte mechanobiology. However, macroscale tools enabling tissue level experiments are needed for a comprehensive understanding at higher organisational levels (Fig. 3). Engineered heart tissues (EHTs) have emerged as a physiological in vitro platform for heart research that is now widely in use. EHTs are 3D cardiac tissue-like structures able to generate contractile force. These commonly used platforms are produced in different shapes from different cells, such as neonatal cardiomyocytes or stem cell-derived cardiomyocytes, which are undergoing either general or chamber specific differentiation programmes (Breckwoldt et al. 2017; Eschenhagen et al. 2002; Goldfracht et al. 2020; Lemme et al. 2018; Schaaf et al. 2011). Mixed with extracellular matrices, for instance, collagen or fibrinogen, heart tissues mature and allow for the analysis of contractile force in dependence of mutations (e.g. ANKRD1 or alpha-actinin 2) (Crocini et al. 2013; Prondzynski et al. 2019), drug treatments (Mannhardt et al. 2016) or presence of additional cell types, such as fibroblasts (Liau et al. 2011), or epicardial cells. In this way, they are used to mimic heart disease or study potential therapies (Bargehr et al. 2019; Hawkes et al. 2019). EHT platforms allow for a range of molecular biology, electrophysiology and force measurement experiments (Goldfracht et al. 2020; Mannhardt et al. 2016; Saleem et al. 2020; Schaaf et al. 2011). Continuous contractile work and tissue spanning between the silicone posts is a probable reason for a good cell alignment and sarcomere organisation based on histological experiments, indicating structural maturation (Schaaf et al. 2011). EHTs have been studies not only as a potential tool for cardiac repair but also for toxicology studies and disease modelling (El-Armouche et al. 2007; Mosqueira et al. 2018; Mühlhäuser et al. 2006; Zimmermann et al. 2006). Work from Xu et al. (2015) presented magnetic actuation as a mechanical stimulator for the microtissue assembled around micropillars, with a potential application to different cell types. In order to study mechano-electrical coupling, Galie et al. (2015) designed a 3D in vitro model of neonatal cardiomyocyte-fibroblast suspended in fibrin-collagen gel (called μTUGs) with incorporated AFM to mechanically control the stimulation. This system allows for direct quantification of contraction velocity and force magnitude, defining properties of striated muscle. Further, Boudou et al. modified previously generated microfabricated tissue gauges (μTUG) for cardiac microtissues (CMTs) that are formed by mixing neonatal cardiomyocytes with collagen-fibrin matrices. This platform allowed to investigate electrical stimulation, mechanical load, matrix stiffness and chemical stimulation on structural and functional properties of CMTs with a potential for further drug screening application (Boudou et al. 2012).

Recent developments are aimed at reducing the size and maturity of the tissues. A mini-EHT model was recently developed, where a reduced cell number (down 16,000 cells compared to ~500,000 cells in normal EHTs) was needed for contractile measurement (Dostanić et al. 2020). To improve maturity, Ronaldson-Bouchard et al. (2018) varied the stimulation frequency during the differentiation, resulting in an adult-like gene expression profile, electrophysiology and tissue ultrastructure. Nunes et al. used both hESC- and hiPSC-derived cardiomyocytes, combining structural and electrical stimulators to generate biowire platforms. Cell suspension in collagen was seeded into the main channel around a suture with progressively increasing electrical stimulation. Biowires exhibited improved organisation, electrophysiological and Ca2+ handling properties when compared to a non-stimulated platform (Nunes et al. 2013).

In addition to EHTs, microfluidic heart-on-a-chip platforms have been developed by different groups to study multicellular microtissues. Ren et al. investigated the H9c2 embryonic cardiomyocyte-like cell line under hypoxic conditions, which showed changes in cell size, mitochondria and suggested more cellular apoptotic events (Ren et al. 2013). The same cell line was also used by Hsiao et al. to investigate hydraulic pressure application, which resulted in bigger cell size and higher levels of natriuretic peptide. This effect could be reversed by a focal adhesion kinase blocker (Hsiao et al. 2015). A microfluidic high-throughput platform incorporating microtissues from small cell numbers (~5000 cells per tissue) and pneumatic mechanical stimulation was developed as a model for cardiac hypertrophy with volume overload and recapitulated the upregulation of the foetal gene programme, associated with this pathology (Parsa et al. 2017).

Finally, cardiac slices emerged as a good model to bridge the gap between in vitro and in vivo models (Pitoulis et al. 2021; Watson et al. 2019). These ultrathin 100–400 μm slices of living myocardium largely maintain tissue architecture, multicellular structure, physiology and load-induced remodelling and hence are an especially useful tool for cardiovascular research, at least until a similarly high degree of maturity can be achieved with in vitro models.

## Conclusions

Cardiovascular diseases (CVD) are still the leading cause of death globally, estimated to reach 17.9 million deaths per year (World Health Organization 2021). Heart failure (HF) is most prevalent among all CVD diseases, whereby around 50% of the cases are identified as HF with reduced ejection fraction and the other half as HF with preserved ejection fraction (HFpEF). To date, there is no effective treatment for HFpEF. With the number of cases rising, this heart disease is posing a significant health burden. However, HF is just at the top of a long list of other heart diseases that need extensive research (Virani et al. 2020). Therefore, studying CVD is of utmost importance. A better understanding of the behaviour of cardiac pathologies is needed but, due to the complexity, requires a multiscale approach to get to the heart of the matter.

The collection of newly developed techniques and tools led to an increasing understanding of cardiomyocyte behaviour and function — temporally and spatially, at different scales. Applying these techniques led to new insights into cardiomyocyte structural properties, electrophysiology and mechanobiology and showed potential to be used as future drug screening platforms. Importantly, they also further established mechanobiology as an integral and crucial part of cardiomyocyte biology. Further interdisciplinary approaches and studies, spanning the different scales, will help to address open questions in cardiovascular research, including the identification of novel therapies for heart failure or how the mechanical environment affects pluripotent stem cells maturation for improved patient-specific models and personalized medicine approaches.

## Data Availability

Not applicable.
